# Supplementary material for: TRAIL-Based Therapies Efficacy in Pediatric Bone Tumors Models Is Modulated by TRAIL Non-Apoptotic Pathway Activation via RIPK1 Recruitment
Source: Cancers (Basel). 2022 Nov 16;14(22):5627. doi: 10.3390/cancers14225627 (PMC9688679; doi:10.3390/cancers14225627)
Supplement: Supplementary file 1 [file cancers-14-05627-s001.zip › cancers-1957629-supplementary.pdf]

cancers

MDPI

Supplementary Materials

TRAIL-Based Therapies Efficacy in Pediatric Bone Tumors Models Is Modulated by TRAIL Non-Apoptotic Pathway Activation via RIPK1 Recruitment

Supplementary methods

Quantitative RT-PCR

Total RNA was extracted from cultured K-HOS cells, parental or previously transduced with shRNA, using a Direct-zol™ RNA MiniPrep Kit (Zymo Research). Two µg of total RNA were retrotranscribed using the Maxima H Minus First Strand cDNA Synthesis Kit (Life Technology). Real-time monitoring of complementary DNA (cDNA) PCR amplification was performed using DNA primers on CFX96 (Bio-Rad Laboratories) with SYBRGreen detection according to the manufacturer's recommendations. PCR amplifications involved 39 cycles of 30 sec at 98°C, 15 sec at 95°C and 30 sec at 60°C. The expression of RIPK1 gene was normalized to that of the endogenous control glyceraldehyde 3-phosphate dehydrogenase (GAPDH). The $2^{-\Delta\Delta Ct}$ (fold change) method was used to calculate expression levels relative to parental cells. Primer sequences: GAPDH: Forward: AAGAACGTGAAGCTCCCTGA, Reverse: AATATAGGGGATGGGCTTGG; RIPK1: Forward: GAGCACCCACAAGAAGAGAA, Reverse: CAGTGCCTTTTCCCTCTGTA.

Supplementary Tables

Table S1. Primary antibodies used for Western blotting

Target	Source	Specie	Cat. N°
Caspase-3	Cell signaling Technology	Rabbit	# 14220
Caspase-8	Cell signaling Technology	Rabbit	# 4790
PARP	Cell signaling Technology	Rabbit	# 9542
Vinculin	Cell signaling Technology	Rabbit	# 13901
phospho- IκBα (Ser32/36)	Cell signaling Technology	Mouse	# 9246
IκBα total	Cell signaling Technology	Rabbit	# 4812
phospho-c-Jun (Ser73)	Cell signaling Technology	Rabbit	# 3270
c-Jun total	Cell signaling Technology	Rabbit	# 9165
RIPK1	Cell signaling Technology	Rabbit	# 4926
DR5	Cell signaling Technology	Rabbit	# 69400

Table S2. Molecular weights

	kDa	$g{\cdot}mol^{-1}$
rhTRAIL	21	21 000.00
AMG655	145	145 645.66
APG880	168	167 785.23

Table S3. IC50 conversion in nM

	rhTRAIL IC50		AMG655 IC50		APG880 IC50	
	ng·mL^{-1}	nM	ng·mL^{-1}	nM	ng·mL^{-1}	nM
K-HOS	>1000	>50	>1000	>7	37	0.22
Saos-2	>1000	>50	>1000	>7		
U-2 OS	>1000	>50	836	5,74		
MG-63	861	41	412	2.83		
EW-24	>1000	>50	>1000	>7		
SK-ES-1	633	30.1	591	4.06		
A-673	198	9.43	481	3.3		
TC-71	85	4.05	143	0.98		

Supplementary Figures

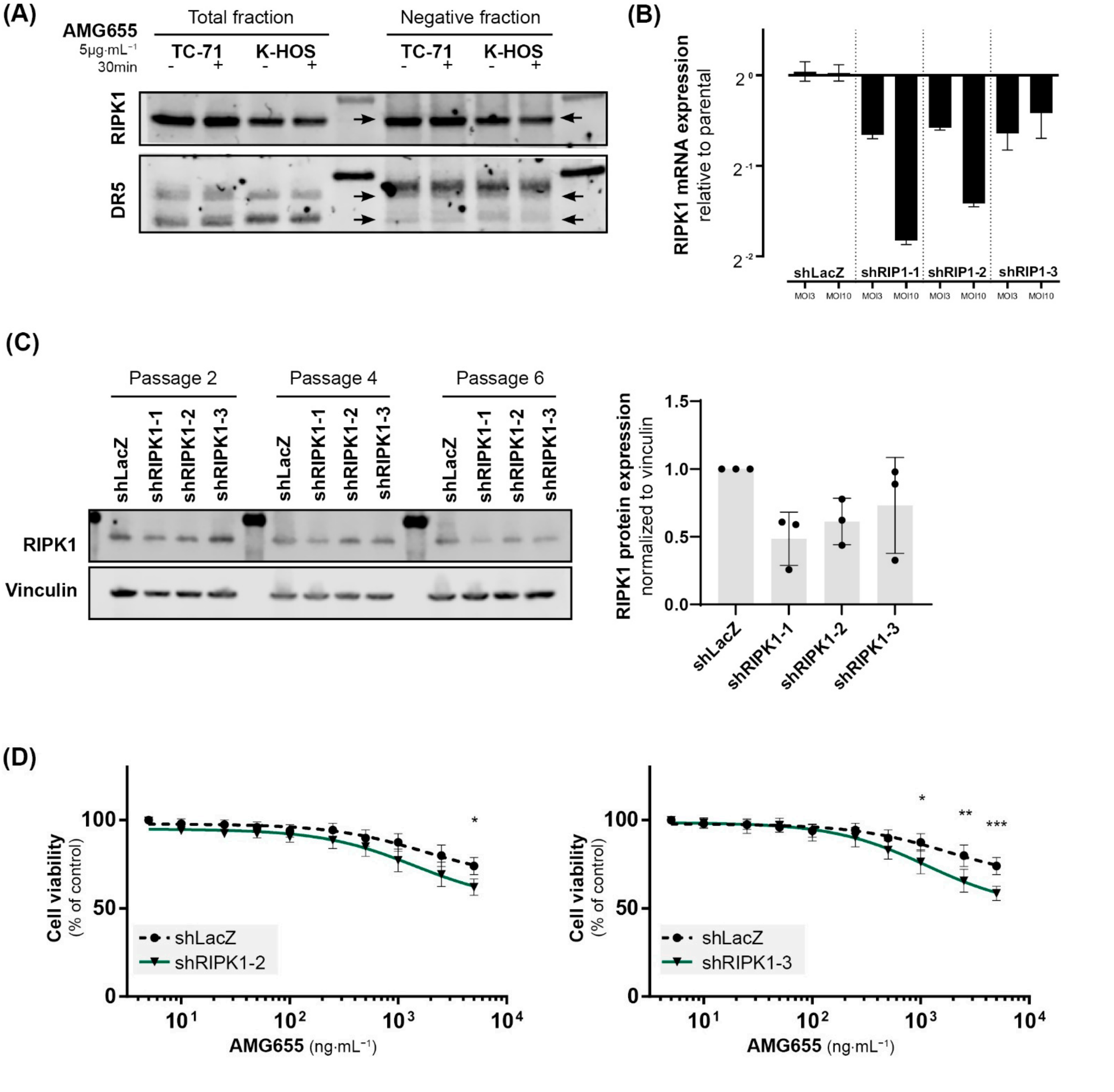

Figure S1. **Validation of RIPK1 inhibition by shRNA targeting**. (**A**) Total fraction and negative fraction of the immunoprecipitation of DR5 using AMG655 antibody at 0 min and 30 min after treatment with 5µg·mL^{-1} of AMG655 on TC-71 and K-HOS cells. Validation of RIPK1 knock-down by shRNA strategy: (**B**) by comparison of RIPK1 gene expression in K-HOS cells parental or transduced with shLacZ, shRIPK1-1, shRIPK1-2 or shRIPK1-3, at multiplicity of infection (MOI) 3 and 10, normalized to GAPDH expression; and (**C**) by Western Blot across several passages. Quantifications of protein expression normalized to Vinculin expression on the right. (**D**) AMG655 sensitivity of shRIPK1-2 and shRIPK1-3 expressing cells was determined by crystal violet staining in the presence of AMG655 compared to control K-HOS cells expressing shLacZ. (* $p < 0.01$, ** $p < 0.05$, *** $p < 0.001$)

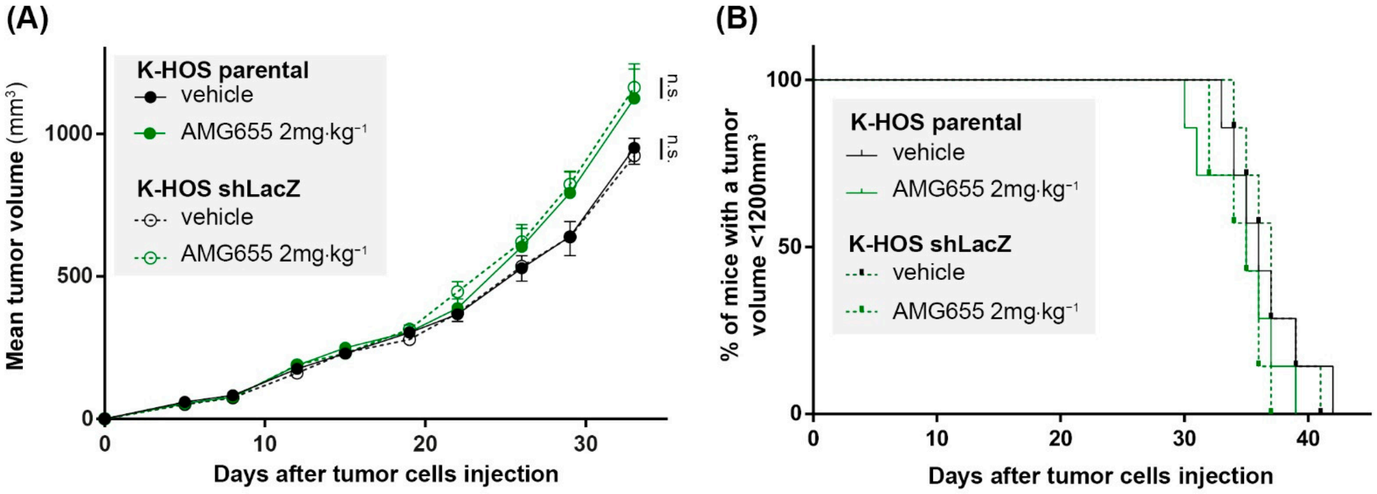

Figure S2. In vivo, the expression of shRNA LacZ does not change K-HOS cell-induced tumors growth in absence or in presence of AMG655. Four groups of seven mice were studied: two groups received paratibial injections of 1 × 10^6 K-HOS parental cells and two groups 1 × 10^6 K-HOS shLacZ cells. For each type of cells, one group of mice was injected twice a week with 0.9% NaCl (control group) and the other group with 2mg·kg^{-1} of AMG655. (**A**) Mean tumor volume per group calculated twice a week. (**B**) Survival evaluated by the percentage of mice with a tumor volume less than 1200mm^3. (n.s.: non-significant)
